# Long noncoding RNAs: functions and mechanisms in colon cancer

**DOI:** 10.1186/s12943-020-01287-2

**Published:** 2020-11-28

**Authors:** Sian Chen, Xian Shen

**Affiliations:** Department of Gastrointestinal Surgery, the Second Affiliated Hospital of Wenzhou Medical University, No 109 Xueyuan West Road, Wenzhou, Zhejiang, 325027 China

**Keywords:** LncRNAs, Colon cancer, miRNAs, Proliferation, Therapy

## Abstract

Evidence indicates that long non-coding RNAs (lncRNAs) play a crucial role in the carcinogenesis and progression of a wide variety of human malignancies including colon cancer. In this review, we describe the functions and mechanisms of lncRNAs involved in colon oncogenesis, such as HOTAIR, PVT1, H19, MALAT1, SNHG1, SNHG7, SNHG15, TUG1, XIST, ROR and ZEB1-AS1. We summarize the roles of lncRNAs in regulating cell proliferation, cell apoptotic death, the cell cycle, cell migrative and invasive ability, epithelial-mesenchymal transition (EMT), cancer stem cells and drug resistance in colon cancer. In addition, we briefly highlight the functions of circRNAs in colon tumorigenesis and progression, including circPPP1R12A, circPIP5K1A, circCTIC1, circ_0001313, circRNA_104916 and circRNA-ACAP2. This review provides the rationale for anticancer therapy via modulation of lncRNAs and circular RNAs (circRNAs) in colon carcinoma.

## Introduction

Colon cancer is one of the most commonly diagnosed digestive cancers worldwide. In America, colon cancer has the fourth highest incidence behind breast, lung and prostate cancers, and is the second leading cause of death after lung cancer [1]. It is estimated that there are more than 1.5 million patients with colorectal cancer (CRC) in America, and 104,610 new cases will be expected in 2020 [1, 2]. In China, CRC is one of the top five diagnosed cancers and causes of cancer-related deaths [3]. Widespread colonoscopy testing has reduced the incidence rate of CRC. Due to improvements in treatments, including colectomy, chemotherapy and immunotherapy, the overall 5-year relative survival rate for colon cancer patients is approximately 64% [2]. Although diet, microorganisms and their metabolites are associated with colon carcinogenesis, the detailed mechanisms of CRC development remain unclear [4]. Therefore, elucidating the molecular mechanisms of colon oncogenesis is of crucial importance.

In recent years, noncoding RNAs (ncRNAs) have been demonstrated to be involved in colon cancer development and progression [5, 6]. It is well known that ncRNAs belong to a class of transcripts that are mostly translated into proteins, but they also play important roles in a variety of cellular and physiologic processes [7]. Long non-coding RNAs (LncRNAs) with a length longer than 200 nucleotides participates in multiple biological processes, including cell proliferation, differentiation, development, apoptosis and metastasis, often by serving as a competing endogenous RNA (ceRNA) to regulate the expression of specific miRNAs, and then target molecules downstream of these miRNAs [8]. In fact, lncRNAs can interact with RNA, DNA and protein, and form RNA-RNA, RNA-DNA, RNA-protein complexes, leading to regulation of gene expression via multiple mechanisms, including modulation of transcription, mRNA stability and translation [9, 10]. LncRNAs can act as a guide, scaffolds or decoy molecule of proteins to recruit proteins or RNAs. LncRNAs can also affect the structure of chromatin and lead to modulating gene expression [11]. In addition, circular RNAs (circRNAs) belong to a new type of ncRNA with a circular configuration and are involved in carcinogenesis [12]. CircRNAs can not only act as sponges for miRNAs and RNA binding proteins, but also serve as mRNA transcriptional regulators and templates for protein translation [13–15]. LncRNAs and circRNAs have been revealed to be associated with the development and progression of a variety of human malignancies including colon cancer [5, 6, 16]. In this review, we will summarize the functions and mechanisms of lncRNAs and circRNAs in human colon oncogenesis and malignant progression.

### Role of lncRNAs in colon cancer

**Fig. 1:**
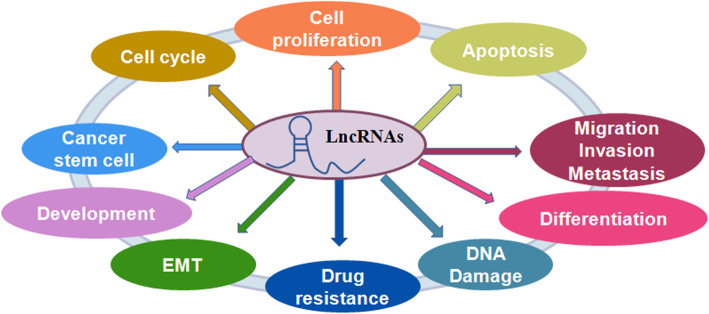
The role of lncRNAs in regulating cellular processes. LncRNAs play a critical role in the regulation of cell proliferation, cell apoptotic death, cell cycle, cell migration and invasion, epithelial-mesenchymal transition (EMT), cancer stem cells, DNA damage and drug resistance in colon cancer

Emerging evidence has implicated that lncRNAs play vital roles in colon carcinogenesis and progression [17, 18], with one study identifying approximately 200 differentially expressed lncRNAs in colon tumors using RNA sequencing data from TCGA dataset [19]. LncRNAs are involved in patient outcome [20], cell proliferation, [21], cell apoptosis [22], cell metastasis and invasion [23], cell cycle [24], epithelial-mesenchymal transition (EMT), cancer stem cells (CSCs) and drug resistance (Fig. 1). In the following section, we will describe the roles of lncRNAs in regulating these cellular processes and highlight the involved molecular mechanisms of lncRNAs (Table 1).

**Table 1:** Representative lncRNAs and related signaling pathways in colon cancer

lncRNA	Expression	Functions	Downstream targets	References
ATB	↑	Enhances invasion, induces EMT	E-cadherin, ZO-1, ZEB1, N-cadherin	[25]
BC200	↑	Increases proliferation, invasion, EMT, inhibits apoptosis, regulates cell cycle	STAT3, β-catenin	[24]
B3GALT5-AS1	↓	Inhibits proliferation, promotes migration, invasion, induces EMT	miR-203, ZEB2, Snail	[26]
CASC15	↑	Promotes proliferation, migration, invasion	miR-4310, LGR5, Wnt/β-catenin	[27]
CASC19	↑	Increases migration	N/A	[28]
CCAT1	↑	Promotes proliferation, invasion, drug resistance	c-Myc	[29–31]
CCAT2	↑	Enhances growth, metastasis	miR-145, WNT	[32, 33]
CYTOR	↑	Promotes migration, invasion, EMT	β-catenin/TCF complex,	[34, 35]
DACOR1	↓	Inhibits proliferation, increases DNA methylation	Cystathionine β-synthase	[36, 37]
DMTF1v4	↑	Increases proliferation, migration, inhibits apoptosis	p-ERK, p-JNK, p-p38	[38]
ENST00000455974	↑	Increases proliferation, migration	JAG2	[39]
FAL1	↑	Enhances proliferation, invasion, Inhibits apoptosis	STAT3, TGF-β1, Bcl-2, p65, PCNA	[40]
FAM83H-AS1	↑	Promotes tumorigenesis	TGF-β signaling	[41]
FER1L4	↓	Inhibits proliferation, migration, invasion	miR-106a-5p	[42]
GSEC	↑	Promotes migration	DHX36	[43]
HNF1A-AS1	↑	Enhances proliferation, migration, invasion	miR-34a/p53	[44]
HOTAIR	↑	Increases migration, invasion	E-cadherin, vimentin, MMP-9	[45–47]
HULC	↑	Promotes proliferation, migration, invasion	miR-613, RTKN, vimentin, N-cadherin, E-cadherin	[48]
H19	↑	Enhances invasion, migration, drug resistance	miR138, HMGA1, miR-675-5p	[49, 50]
Linc00973	/	Involves in drug resistance	N/A	[51]
Linc01106	↑	Confers proliferation, migration, stemness	miR-449b-5p, Gli	[52]
Linc01234	↑	Promotes proliferation	miR-642a-5p, SHMT2	[53]
Linc01567	↑	Promotes proliferation, invasion, migration	miR-93	[54]
Linc00261	↓	Inhibits proliferation, invasion, migration, drug resistance, induces apoptosis.	β-catenin, Wnt pathway	[55]
Linc00657	↓	Inhibits proliferation, invasion, induces apoptosis	CAPN7, PI3K/Akt	[56]
Linc01082	↓	Represses proliferation, migration, Invasion	/	[21]
Linc01578	↑	Enhances metastasis	NF-κB, YY1	[57]
Linc02418	↑	Promotes growth, mobility, invasion, inhibits apoptosis	miR-34b-5p, Bcl-2	[58]
Lnc34a	↑	Enhances proliferation, stem cells	Dnmt3a, PHB2,HDAC1	[59]
Loc285194	↓	Inhibits proliferation	miR-211	[60]
Loc441461	↑	Increases growth, motility, inhibits apoptosis	PhoA, ROCK	[61]
LincDUSP	↑	Promotes proliferation, stem cells, modulates DNA damage response and cell cycle, inhibits apoptosis	ATR, p53, E2F, c-Myc	[19]
MALAT1	↑	Enhances proliferation, invasion, migration	miR-129-5p, HMGB1, miR-663a	[62–64]
MYU	↑	Increases proliferation, controls cell cycle	hnRNP-K, CDK6	[65]
PCAT6	↑	Promotes proliferation, induces apoptosis	EZH2, H3K4me3	[22]
Pint	↓	Inhibits proliferation	PRC2	[66]
PVT1	↑	Promotes proliferation, migration, invasion	miR-26b, miR-30d-5p/RUNX2	[67, 68]
RBM5-AS1	/	Promotes proliferation, maintains stem cells	WNT, β-catenin	[69]
ROR	↑	Enhances proliferation, migration, invasion	miR-145	[70]
RP11-619 L19.2	↑	Enhances proliferation, migration, invasion, EMT	miR-1271-5p, CD164	[71]
SnaR	↑	Promotes drug resistance	N/A	[72]
SBDSP1	↑	Promotes proliferation, migration, invasion, regulates cell cycle,	p21, cyclin D1, Akt, ERK1/2, STAT3	[73, 74]
SNHG1	↑	Increases proliferation, invasion, migration, inhibits apoptosis	Wnt/β-catenin, c-Myc, cyclin D	[75]
SNHG7	↑	Increases proliferation, inhibits apoptosis	miR-193b, K-ras/ERK/cyclinD1	[76]
SNHG15	↑	Enhances proliferation, migration	Slug	[77]
SNHG17	↑	Elevates proliferation, migration, invasion	miR-375, CBX3	[78]
TUG1	↑	Promotes proliferation, migration, invasion, EMT, inhibits apoptosis,	miR-26a-5p, MMP-14, VEGF, MAPK, Hsp27	[23, 79]
UPAT	↑	Promotes survival	UHRF1	[80]
USP2-AS1	↑	Enhances proliferation, metastasis	Phosph-YAP	[81]
XIST	↑	Promotes proliferation	miR-34a, Wnt, β-catenin cyclin D1, c-Myc, MMP-7	[82]
ZEB1-AS1	↑	Enhances proliferation, migration, invasion	miR-455-3p, PAK2	[20]
ZFAS1	↑	Increases proliferation, invasion, EMT, inhibits apoptosis,	ZEB1, E-cadherin, ZO-1, vimentin, N-cadherin	[83]

#### LncRNAs are associated with patient outcomes

Aberrant expression of lncRNAs has been reported to be correlated with the clinicopathological parameters of colon cancer [84]. For example, lncRNA zinc finger E-box-binding homeobox 1 antisense 1 (ZEB1-AS1) expression is significantly elevated in colon adenocarcinoma tissues, which is consistent with data from TCGA database set [20]. Higher ZEB1-AS1 expression is observed in colon adenocarcinoma cell lines, including SW480, HT29, LS174T, HCT116 and DLD-1, when compared with normal colon histiocytes [20]. Increased ZEB1-AS1 is associated with an advanced stage, lymph node metastasis and distant metastasis in colon cancer patients. Moreover, patients with ZEB1-AS1 upregulation have poor survival [20]. FAM83H-AS1, one of dysregulated lncRNAs in several cancers, was shown to be highly expressed in colon cancer patients by RNAscope in situ hybridization analysis, and is associated with shorter overall survival time [41]. Moreover, FAM83H-AS1 was observed to be negatively correlated with Smad1/5/9 levels, key factors in TGF-β signaling, in colon cancer specimens [41]. The results of this study indicate that FAM83H-AS1 functions in part via the regulation of TGF-β signaling in colon cancer [41]. High LINC01296 expression has been observed in colon cancer tumor tissues, which is also correlated with advanced Dukes’ stage, poor prognosis and shorter survival rate [85]. Similarly, LINC01234 was reported to be highly expressed in colon cancer tissues, and colon cancer patients with upregulated LINC01234 have a shorter survival time [53]. ENST00000455974 expression is correlated with TNM stage and distant metastasis in colon cancer with DNA mismatch repair-proficient. Moreover, ENST00000455974 levels increase in the progression from normal colon, adenoma, carcinoma, and metastatic colon cancer tissues. Furthermore, higher expression of ENST00000455974 is related with shorter survival time in colon cancer patients [39]. Prostate cancer-associated ncRNA transcript 6 (PCAT6) is overexpressed in colon cancer tissues and is associated with advanced clinical stages and a worse prognosis [22].

Cytoskeleton regulator RNA (CYTOR), also named Linc00152, is upregulated in colon cancer patients and may be a predictive factor of a worse prognosis [34, 35]. CYTOR expression levels are correlated with TNM stage, disease-free survival and overall survival rates in patients with colon cancer [34]. LncRNA plasmacytoma variant translocation 1 (PVT1) expression is remarkably increased in colon cancer tissues and correlates with lymph node metastasis, clinical stages and survival time in patients with colon cancer [67]. Focally amplified lncRNA on chromosome 1 (FAL1) expression is strikingly increased in colon cancer specimens and is associated with a lower survival rate in colon cancer patients, indicating that FAL1 may be an independent prognostic factor in this disease [40]. Higher lncRNA BC200 expression was reported in colon tumor tissues and shown to be associated with TNM stage and lower survival time in patients with colon cancer [24]. Another lncRNA, HOTAIR, was observed to be highly expressed in colon cancer tissues and correlated with tumor invasion, metastasis, tumor stage and survival time [45–47]. The expression of lncRNA cancer susceptibility 15 (CASC15) is increased in colon tumor tissues and associates with TNM stage and tumor metastasis [27]. Small nucleolar RNA host gene 15 (SNHG15) is also upregulated in colon cancer patients and correlates with poor prognosis [77]. One group showed that lncRNA SBDSP1 is elevated in colon cancer tissues and further correlates with differentiation, invasion depth, TNM stage, and survival time in colon cancer patients [73]. LncRNA zinc finger antisense 1 (ZFAS1) is overexpressed in colon cancer specimens and correlates with TNM stage, vascular invasion and metastasis. Moreover, plasma of colon cancer patients has higher levels of ZFAS1 [83]. HNF1A-antisense 1 (HNF1A-AS1) expression is also higher in colon cancer tumors and correlates with advanced stage, metastasis and survival time in colon cancer patients [44].

LincRNA-ROR levels have been reported to be highly expressed in colon cancer tissues and are associated with tumor invasion and lymph node metastasis and AJCC stage in colon cancer patients [70]. LncRNA-activated by TGF-beta (lncRNA-ATB) expression is elevated in colon cancer tissues, especially in metastatic cancer specimens. LncRNA-ATB levels are associated with AJCC stage and survival time in patients with colon cancer [25]. In addition, lncRNA highly upregulated in liver cancer (HULC) is promoted in colon cancer tissues and correlates with clinicopathological parameters, including tumor stage, tumor size, metastasis and survival rate. CCAT1 expression has also been reported to be highly increased in colon cancer tissues and is associated with clinical stage, lymph nodes metastasis, and survival time [29]. Increased CASC19 expression is observed in colon cancer tissues and is associated with tumor size and metastasis [28]. Accumulating evidence has dissected that lncRNAs also perform antitumor activities in colon cancer. For example, B3GALT5-AS1 is decreased in colon tumor specimens, especially in liver metastasis tissues [26]. Moreover, lower expression of B3GALT5-AS1 levels is correlated with liver metastasis and poor outcome in colon cancer patients [26]. Similarly, LINC00657 expression is reduced in colon cancer patients, especially in patients with distant metastasis [56]. Lower LINC00657 expression is associated with TNM stage, tumor size and prognosis in colon cancer patients [56]. Fer-1-like protein 4 (FER1L4) levels are reduced in colon tumor specimens and is negatively associated with tumor invasion, metastasis and tumor stage, and colon cancer patients with lower FER1L4 expression have worse overall survival and disease-free survival rates [42].

#### LncRNAs regulate cell proliferation

Increasing evidence has revealed that lncRNAs play an important role in the regulation of cell proliferation. Multiple studies have clarified that lncRNA dysregulation is involved in governing colon cancer cell proliferation. ZEB1-AS1 overexpression facilitates cell growth by promoting p21-activated kinases 2 (PAK2) expression by sponging miR-455-3p in colon adenocarcinoma cells [20]. LINC01082 is downregulated in colon cancer tissues, and LINC01082 upregulation inhibits cell proliferation in SW480 and SW620 colon cancer cells [21]. LINC01296 silencing was found to suppress cell proliferation by targeting miR-21a in colon cancer cells [85]. Higher taurine-upregulated gene 1 (TUG1) expression has been reported in colon cancer tissues, and p63 downregulation increases TUG1 expression in HCT116 and LoVo colon cancer cells [79]. Moreover, knockdown of TUG1 suppresses the proliferation of HCT116 and LoVo cells [79]. Similarly, another study identified that knockdown of TUG1 blocks the proliferation of colon cancer cells, and impedes tumor growth in vivo [23]. Mechanistically, TUG1 overexpression elevates p-p38 mitogen-activated protein kinase (p-p38 MAPK), and p-heat shock protein 27 (p-Hsp27) levels in colon cancer [23]. LncRNA small nucleolar RNA host gene 7 (SNHG7) is overexpressed in colon advanced adenomas and early-stage colon cancer, and SNHG7 downregulation in HT29 cells retards cell proliferation via interacting with miR-193b and suppressing K-ras/ERK/cyclin D1 [76]. Furthermore, LINC01234 enhances colon cancer cell proliferation via the upregulation of serine hydroxymethyltransferase 2 (SHMT2) by competitively binding with miR-642a-59 [53]. Downregulation of ENST00000455974 results in proliferation inhibition of colon cancer cells via the suppression of JAG2 [39]. PCAT6 was reported to increase cell growth by inhibiting colon cancer cell apoptosis [22]. Downregulation of HULC impairs colon cancer cell proliferation in vitro and retards tumor growth in mice by regulation of miR-613/rhotekin (RTKN) [48].

LncRNA DMTF1v4 was reported to be overexpressed in colon cancer tissue, and DMTF1v4 knockdown in HT-29 cells suppresses cell proliferation via inhibition of p-ERK, p-JNK, and p-p38. Notably, knockdown of DMTF1v4 retards colon tumor growth in nude mice [38]. Studies have shown that PVT1 enhances cell growth via inhibiting miR-26b [68], while reduced PVT1 levels attenuates cell growth via regulation of the miR-30d-5p/RUNX2 axis [67]. One group identified that small nucleolar RNA host gene 1 (SNHG1) expression is overexpressed in colon cancer cells and tumor specimens. SNHG1 accelerates cell proliferation via upregulating β-catenin, c-Myc and cyclin D1 protein levels in colon cancer cells [75]. In addition, metastasis associated lung adenocarcinoma transcript 1 (MALAT1) is highly expressed in several colon cancer cell lines such as LoVo, HCT116, SW480, and HT29 compared with normal intestinal epithelial HIEC cells. Downregulation of MALAT1 attenuates SW480 and HCT116 cell proliferation by acting a ceRNA to inhibit high motility group box protein 1 (HMGB1) via sponging miR-129-5p in colon cancer cells [62]. Similarly, another study also validated that MALAT1, which is highly expressed in colon cancer tissues, promotes SW480 and HCT116 colon cancer cell growth via binding miR-663a and upregulating several targets of miR-663, including TGF-β1, PIK3CD, p53, p21, and JUND [63]. Zhang et al. demonstrated that inhibition of FAL1 suppresses cell proliferation, whereas FAL1 upregulation increases colon cancer cell proliferation. Mechanistically, FAL1 interacts with STAT3 and triggers its phosphorylation to modulate the expression of Bcl-2, TGF-β1, p65, and PCNA in colon cancer cells [40]. Knockdown of lncRNA BC200 attenuates HCT-116 and HT29 colon cancer cell proliferation via downregulation of pSTAT3, Ki-67 and PCNA [24]. One study identified that B3GALT5-AS1 reduces colon cancer cell proliferation [26]. LncRNA XIST expression is increased in colon cancer tissues, while XIST silencing inhibits colon cancer cell growth and reduces tumor growth in vivo. Moreover, XIST can bind miR-34a and upregulate the expression of WNT1, β-catenin, cyclin D1, c-Myc and MMP-7 [82]. Upregulation of LINC00657 inhibits cell viability via promoting CAPN7 expression and suppressing the PI3K/Akt pathway in colon cancer cells [56].

In addition, CASC15 knockdown suppresses colon cancer cell proliferation and tumor growth in vitro and in vivo. Moreover, CASC15 serves as a sponge to inhibit miR-4310 and alter leucine-rich repeat-containing G-protein coupled receptor 5 (LGR5) expression, leading to activation of the Wnt/β-catenin signaling pathway [27]. Ectopic SNHG15 expression enhances tumor growth in a colon cancer xenografted model [77]. Downregulation of lincDUSP, an oncogenic lncRNA, inhibits the proliferation of patient-derived colon tumor cells [19]. LncRNA colon cancer-associated transcript-2 (CCAT2) has been shown to harbor the rs6983267 SNP, which can regulate CCAT2 transcription. CCAT2 overexpression has been reported in CRC tissues [32]. Moreover, CCAT2 interacts with TCF7L2 and leads to WNT pathway activation, which subsequently enhances tumor growth and metastasis as well as chromosomal instability [32]. CCAT2 overexpression in HCT116 cells leads to promotion of xenograft tumor formation in nude mice [32]. CCAT2 has been shown to block the maturation of miR-145 by preventing pre-miR-145 export to the cytoplasm from nucleus, controlling colon cancer cell proliferation and differentiation [33]. Knockdown of CCAT2 suppresses HCT-116 cell proliferation, while CCAT2 overexpression facilitates the proliferation [33]. LINC00261 levels were reported to be decreased in colon cancer tissues, and upregulation of LINC00261 was shown to inhibit colon cancer cell viability by blocking β-catenin export to the nucleus from the cytoplasm or by promoting β-catenin degradation [55]. Moreover, ZFAS1 overexpression accelerates colon cancer cell proliferation [83]. Ectopic HNF1A-AS1 expression promotes colon cancer cell viability and tumor growth in vitro and in vivo. Moreover, HNF1A-AS1 silencing retards tumor growth in colon cancer xenograft models. Mechanistically, HNF1A-AS1 functions as a ceRNA to regulate miR-34a, consequently modulating SIRT1/p53 and activation of the Wnt signaling pathway [44]. Additionally, lincRNA-ROR downregulation in PKO colon cancer cells inhibits their proliferation [70]. Lnc34a has been shown to recruit Dnmt3a via PHB2 and HDAC1 to regulate miR-34a levels, leading to the promotion of CRC growth in xenograft mice. In agreement with this finding, Lnc34a is upregulated in late stage CRCs, leading to CRC cell proliferation [59]. FER1L4 overexpression inhibits the proliferation of colon cancer cells by acting as a ceRNA by sponging miR-106a-5p [42].

One study used RIP-sequenced and identified numerous lncRNAs that interact with DNMT1, a DNA methyl transferases, in HCT116 cells. Among these lncRNAs, DNMT1-associated colon cancer repressed lncRNA 1 (DACOR1) levels are decreased in colon tumors and cancer cell lines. Consistent with these findings, overexpression of DACOR1 decreased colony formation in colon cancer cells via activation of anticancer signaling pathways and inactivation of cancer-associated metabolic pathways [36]. Furthermore, c-Myc can promote CCAT1 transcription by directly binding to its promoter region. Ectopic CCAT1 expression stimulates cell proliferation in colon cancer cells [29]. Moreover, c-Myc directly binds to the promoter region of CCAT1 and enhances CCAT1 transcription [29]. One group characterized that lncRNA p53 induces noncoding transcript (Pint), which is governed by p53 pathway, facilitates cell proliferation and survival by modulating the TGF-β, MAPK and p53 pathways. Pint can interact with PRC2 and promotes H3K27 tri-methylation and repression [66]. LncRNA loc285194, a p53 transcript target, exhibits decreased expression in colon tumors, and its overexpression suppresses cancer cell growth in vitro and in vivo via inhibition of miR-211 [60]. LncRNA UPAT interacts with and stabilizes UHRF1 by blocking the β-transducin repeat-containing protein (β-TrCP)-induced ubiquitination of UHRF1 to promote Stearoyl-CoA desaturase 1 and Sprouty 4, of which is necessary to maintain colon cancer cell survival [80]. Taken together, these findings demonstrate that lncRNAs regulate cell proliferation in colon cancer cells.

#### LncRNAs regulate cell apoptosis

LncRNAs have been reported to govern cell apoptosis in colon cancer. In vitro and in vivo experiments have shown that PCAT6 inhibits cell apoptosis by promoting the enrichment of EZH2 and H3K4me3 at the ARC region, leading to an increased ARC transcriptional activity in colon cancer cells [22]. The inhibition of SNHG7 in HT29 colon cancer cells stimulates cell apoptosis by inactivating K-ras/ERK/cyclin D1 signaling [76].

DMTF1v4 downregulation induces colon cancer cell apoptosis via upregulating the expression of apoptosis-related proteins [38]. SNHG1 overexpression inhibits colon cancer cell apoptosis possibly via the Wnt/β-catenin signaling pathway [75]. Downregulation of FAL1 in HT29 colon cancer cells stimulates cell apoptosis, indicating that FAL1 may inhibit cell apoptosis and increase cell proliferation in cells [40]. One group reported that silencing of BC200 stimulates the apoptosis of HCT-116 and HT29 colon cancer cells [24]. In addition, overexpression of LINC00657 increases cell apoptosis via inactivation of the PI3K/Akt pathway in colon cancer cells [56]. In another study, downregulation of lincDUSP was shown to induce the apoptosis of patient-derived colon cancer cells [19]. LINC00261 overexpression has been demonstrated to promote cell apoptosis [55], whereas lncRNA ZFAS1 impedes colon cancer cell apoptosis [83]. Moreover, the oncogenic lncRNA TUG1 inhibits the apoptosis of HCT116 and LoVo colon cancer cells [79].

#### LncRNAs regulate invasion and metastasis

LncRNAs have been shown to have crucial roles in regulating the migration, invasion and metastasis of colon cancer cells. ZEB1-AS1 facilitates cell invasion and migration via increasing PAK2 expression by sponging miR-455-3p, leading to the metastasis of colon cancer cells [20]. Upregulation of LINC01082 inhibits the migration and invasion of SW480 and SW620 colon cancer cells, indicating that LINC01082 has antitumor activity in colon cancer [21]. One study showed that LINC01296 downregulation retards the invasive activity of colon cancer cells by targeting miR-21a [85]. In addition, knockdown of TUG1 expression has been shown to attenuate the migration of HCT116 and LoVo colon cancer cells [79]. LncRNA HULC knockdown inhibits cell migration and invasion by modulating miR-613/RTKN expression in colon cancer cells [48]. Silencing of TUG1 impedes cell invasion in vitro and in mice via regulation of the miR-26a-5p/MMP14/p38 MAPK/Hsp27 axis [23]. Inhibition of ENST00000455974 causes disrupts the migration of colon cancer cells, whereas ENST00000455974 upregulation enhances the metastasis of colon cancer via the upregulation of JAG2 [39]. Downregulation of HULC attenuates the metastasis of colon cancer cells by interacting with miR-613 and modulating RTKN [48]. LncRNA DMTF1v4 overexpression enhances the migration of HT-29 cells via regulating the ERK/MAPK signaling pathway in colon cancer cells [38]. In another study, PVT1 was reported to enhance cell migration and invasion via the modulation of miR-26b and miR-30d-5p in colon cancer cells [67, 68].

One study demonstrated that inhibition of CYTOR retards the migration and invasion of colon cancer cells, while overexpression of CYTOR promotes colon cancer cell metastasis. Mechanistically, CYTOR enhances cell invasion and metastatic properties by interacting with β-catenin in colon cancer [34]. SNHG1 upregulation elevates cell migration and invasion via regulation of the Wnt/β-catenin signaling pathway [75]. In vitro invasion assay results demonstrated that FAL1 increases the invasion in human colon cancer cells. Notably, FAL1 promotes cell invasion in part via modulation of Bcl-2, TGF-β1, p65, and PCNA expression in colon cancer cells [40]. Knockdown of BC200 blocks the invasion of HCT-116 and HT29 colon cancer cells via downregulation of MMP-2 and MMP-9 [24]. In another study, B3GALT5-AS1 was shown to increase colon cancer cell migration and invasion by binding to the miR-203 promoter and suppressing miR-203 expression. The results of an in vivo study also demonstrated that B3GALT5-AS1 attenuates the liver metastasis of colon cancer cells via the repression of miR-203 [26]. In another study, LINC00657 overexpression was shown to increase invasive ability of colon cancer cells via targeting the PI3K/Akt pathway and CAPN7 expression [56].

LncRNA MALAT1 activation promotes cell migration and invasion in SW480 and HCT116 cells by binding to miR-663a and promoting the activation of its targets [62]. Interestingly, one study reported that MALAT1 inhibits colon cancer cell migration and invasion via regulation of EpCAM and ITGB4. Moreover, PTEN controls MALAT1 expression via sponging several oncogenic miRNAs, including miR-17, miR-20a, and miR-106b [64]. In addition, CASC15 inhibition reduces the migration and invasion of colon cancer cells via regulation of the miR-4310/LGR5/Wnt/β-catenin axis [27]. SNHG15 overexpression accelerates the migration of colon cancer cells via interaction with the Slug domain, which impairs the ubiquitination and degradation of Slug and maintains its stability in colon cancer [77]. LncRNA H19 is upregulated in colon cancer tissues, and its knockdown represses the migratory and invasive ability of colon cancer cells via sponging miR-138 and promoting the subsequent upregulation of high-mobility group A (HMGA1) [49]. LINC00261 upregulation attenuates colon cancer cell migrative and invasive ability via regulation the WNT/β-catenin pathway [55]. Downregulation of CASC19 blocks the migratory ability of CRC cells, indicating that CASC19 may enhance CRC metastasis [28]. G-Quadruplex-forming sequence containing lncRNA (GSEC) was reported to be upregulated in colorectal cancer tissues, and its downregulation represses cell motility via suppressing DHX36 function in colon cancer [43]. LncRNA ZFAS1 promotes cell invasion in colon cancer cells [83]. Overexpression of HNF1A-AS1 increases colon cancer cell migration and invasion, while its downregulation attenuates tumor metastasis in colon cancer xenograft mice via regulation of the miR-34a/p53 signaling axis [44]. The downregulation of lincRNA-ROR in RKO colon cancer cells impedes their migratory and invasive ability by altering the activity of miR-145 [70]. LncRNA-ATB expression levels are higher in three highly invasive colon cancer cell lines, including Caco205, SW620 and Lovo cells than in the three poorly invasive cell lines SW480, Caco2 and HCT116 [25]. FER1L4 expression is decreased in colon cancer tissues and its overexpression impairs cell migration and invasion via downregulation of miR-106a-5p in colon cancer cells [42]. HOTAIR facilitates the migration and invasion of colon cancer cells in part via regulation of MMP-9 [46]. LncRNA CCAT1 increases cell invasion in colon cancer cells, which is regulated by c-Myc via binding to the CCAT1 promoter and activating its transcription [29]. Specifically, CCAT2-overexpressing HCT116 cells exhibit liver metastasis in nude mice. Consistent with these results, CCAT2 knockdown attenuates the invasiveness of KM12SM colon cancer cells [32]. Silencing of SBDSP1 represses proliferation, migration, and invasion and induces G0/G1 cell cycle arrest via downregulation of p21, cyclin D1, Akt, ERK1/2, and STAT3 phosphorylation in colorectal cancer [74]. LncRNA SNHG17 elevates proliferation, migration, and invasion via serving as miR-375 sponge to control the expression of chromobox 3 (CBX3) in colon cancer [78]. Linc02418 promotes growth, mobility and invasion and inhibits apoptosis via targeting miR-34b-5p and Bcl-2 pathways in colon cancer [58]. LncRNA USP2-AS1 enhances cell proliferation and metastasis via downregulation of phosph-YAP in colon cancer [81]. LncRNA LOC441461 knockdown suppresses growth and induces apoptosis and represses motility via the inhibition of PhoA/ROCK pathways in colon cancer [61]. Linc01578 enhances tumor metastasis via modulation of NF-κB and Yin Yang 1 (YY1) axis in colon cancer [57].

#### LncRNAs regulate the cell cycle

LncRNAs have been revealed to control cell cycle of colon cancer cells. For instance, lncRNA BC200 silencing induces cell cycle arrest at G0/G1 phase, possibly by reducing the expression of cyclin D1, cyclin E, and c-Myc through decreasing β-catenin levels [24]. Downregulation of lincDUSP induces cell cycle arrest at S phase in colon cancer cells potentially via regulation of cell cycle control pathways [19]. The levels of lncRNA MYU, a downstream target of c-Myc, are increased in most colon cancers and enhance the G1-S transition in the cell cycle process via its interaction with hnRNP-K and subsequent stabilization of CDK6 in colon cancer cells. Furthermore, lncRNA MYU promotes the proliferation and tumorigenicity of CRC cells [65].

#### LncRNAs regulate cancer stem cells

Emerging evidence has supported that CSCs play a crucial role in tumorigenesis, metastasis, recurrence and drug resistance. LncRNAs have been validated to participate in CSCs formation and maintenance in human cancers, including CRC. Lnc34a is overexpressed in colon CSCs and can trigger asymmetric division and promote the self-renewal of colon CSCs [59]. Increased lncRNA RBM5-AS1 expression is observed in colon cancer stem-like cells, with RBM5-AS1 promoting cell growth and survival via activation of the WNT signaling pathway through its interaction with β-catenin. Thus, RBM5-AS1 is important for the maintenance of colon CSCs [69]. Overexpression of CCAT2 increases the levels of multiple molecular markers of CSCs in colon cancer cells, indicating that CCAT2 may be involved in contributing to CSCs development [33]. LINC01567 (also known as LOCCS) is highly expressed in colon CSCs that express CD133+/CD166+/CD44+, and its inhibition suppresses the proliferation, migration, invasion and tumor xenografts of colon CSCs via sponging miR-93 [54]. Recently, linc01106 confers colon cancer proliferation, migration, and stemness via regulating the Gli family factors partly by sponging miR-449b-5p [52].

#### LncRNAs regulates the EMT process

EMT is a cellular process in which epithelial cells are changed to mesenchymal cells, providing them the ability to migrate and metastasize. Downregulation of TUG1 was shown to suppress EMT in part by targeting miR-26a-5p and MMP-14, VEGF, MAPK and Hsp27 in colon cancer cells [23]. The results of this study suggest that TUG1 facilitates EMT in colon cancer cells. The loss of epithelial characteristics and the simultaneous gain of mesenchymal features are correlated with CYTOR expression, the upregulation of which triggers EMT in colon cancer cells [34]. Consistently, CYTOR expression is associated with the loss of epithelial features and the acquisition of mesenchymal characteristics in multiple colon cancer cell lines [34]. Importantly, BC200 downregulation inhibits EMT in HCT-116 and HT29 colon cancer cells in part via the suppression of STAT3 phosphorylation [24].

B3GALT5-AS1 induces EMT due to the suppression of miR-203 and the subsequent upregulation of miR-203 downstream targets such as ZEB2 and Snail2 in colon cancer cells, leading to colon cancer liver metastasis [26]. LncRNA ZFAS1 accelerates the EMT process via the induction of ZEB1 expression, while knockdown of ZFAS1 upregulates the expression of E-cadherin and ZO-1, but decreases that of ZEB1, vimentin and N-cadherin in colon cancer cells [83]. LncRNA-ATB downregulation enhances the expression of E-cadherin, ZO-1, while reducing that of ZEB1 and N-cadherin (N-cad), and influences EMT in colon cancer cells [25]. Silencing HOTAIR elevates E-cadherin expression and suppresses that of vimentin and MMP-9, which can lead to EMT in colon cancer cells [46]. LncRNA HULC knockdown leads to a decrease in the expression of N-cadherin and vimentin, and an increased E-cadherin expression [48], indicating that HULC may be involved in the EMT process in colon cancer cells. Recently, one study showed that downregulation of RP11-619 L19.2 represses cell proliferation migration, invasion and EMT in colon cancer via targeting the miR-1271-5p/CD164 axis [71].

#### LncRNAs regulate drug resistance

Overcoming drug resistance is a major challenge for achieving better outcomes of cancer therapy and lncRNAs have been implicated in drug resistance development. Using RNA-Seq, RT-qPCR, bioinformatics, and murine tumor xenograft models, one group reported that the expression of five lincRNAs, including LINC00973, LINC00941, CASC19, CCAT1 and BCAR4, are consistently altered in HT-29 and HCT-116 cells after treatments with 5-FU, oxaliplatin and irinotecan both in vitro and in vivo. Among these five lincRNAs, LINC00973 is most strongly upregulated in colon cancer cells treated with 5-FU, oxaliplatin, and irinotecan [51]. This report demonstrated that lncRNAs may participate in drug resistance in colon cancer. One study revealed that lncRNA CCAT1 is highly expressed in colon tumor specimens and colon cancer cell lines as well as 5-FU-resistent cells. CCAT1 overexpression in colon cancer cells reduces their sensitivity of 5-FU and promoted decreased apoptosis rates, whereas knockdown of CCAT1 leads to the opposite effects in colon cancer cells [30]. These results indicate that CCAT1 is involved in 5-FU resistance in colon cancer cells.

LncRNA H19 reduces vitamin D receptor (VDR) expression via miR-675-5p, while the VDR pathway also represses H19 expression via regulation of the c-Myc/Mad-1 pathway. Notably, H19 overexpression stimulates resistance to 1,25(OH)2D3 treatment in colon cancer cells and mouse models [50]. LINC00261 downregulation is observed in cisplatin-resistant colon cancer cells, while LINC00261 upregulation reverses cisplatin resistance and enhances the cisplatin effects of this drug via inactivation of the β-catenin/WNT pathway [55]. CYTOR overexpression leads to resistance to oxaliplatin-induced apoptosis in colon cancer cells. CYTOR functions as a ceRNA to regulate miR-193a-3p expression and subsequently upregulates that of Erb-b2 receptor tyrosine kinase 4 (ERBB4). Inhibition of ERBB4 reduces pAkt levels and attenuates oxaliplatin resistance [35]. One study measured the expression pattern of 90 lncRNAs via qPCR-based profiling in colon cancer SNU-C4 and SNU-C5 cells with 5-FU-resistance and identified 19 differentially expressed lncRNAs and 23 lncRNAs in 5-FU-resistent-SNU-C4 and SNU-C5 cells, respectively, including the lncRNA snaR and BACE1AS. Moreover, inhibition of snaR increases cell sensitivity to 5-FU treatment, indicating that snaR contributes to 5-FU resistance in colon cancer cells [72].

#### LncRNAs are involved in DNA methylation and the DNA damage response

DNA methylation has been shown to play a crucial role in chromatin organization and gene expression [86]. DNA methylation occurs in gene promoters, gene bodies and intergenic regions of the genome, and is important in regulating gene expression [86]. Downregulation of lincDUSP in colon cancer cells promotes S-phase accumulation and γH2AX foci formation, suggesting that lincDUSP is involved in inducing DNA damage response [19]. Overexpression of DACOR1 decreases cystathionine β-synthase expression and inhibits the production of S-adenylyl methionine, an important methyl donor for DNA methylation [36]. Therefore, DACOR1 contributes to aberrant DNA methylation and colon carcinogenesis [36]. Moreover, this group demonstrated that DACOR1 upregulation reprograms genome-wide DNA methylation in colon cancer cells, including gene promoters, gene bodies, and intergenic regions, leading to the inhibition of FOS and JUN and the repression of AP-1 activity in colon cancer cells [37].

### Role of circular RNA in colon cancer

Circular RNAs (circRNAs) play essential roles in the development and progression of colon cancer (Table 2). The circRNA circPPP1R12A is highly expressed in colon cancer tissues and its overexpression in colon cancer patients is correlated with shorter overall survival [87]. In addition, the circPPP1R12A-73aa protein, encoded by circPPP1R12A, increases cell proliferation, migrative and invasive abilities via regulation of the Hippo-YAP signaling pathway in colon cancer cells [87]. CircPIP5K1A is overexpressed in colon cancer tissues. Reduced circPIP5K1A expression attenuates cell viability and reduces cell motility, while circPIP5K1A overexpression facilitates colon cancer cell migration and invasion. Mechanistically, enforced expression of circPIP5K1A increases AP-1 expression and alleviates that of CDX-2, Zic-1, and IRF-4 in colon cancer cells in part via the suppression of miR-1273a. Therefore, circPIP5K1A augments colon oncogenesis via inhibiting miR-1273a [88]. Higher circCTIC1 expression is observed in colon tumor tissues and TICs, which is associated with tumor prognosis. Inhibition of circCTIC1 represses the self-renewal of colon TICs, while its upregulation augments TIC self-renewal via c-Myc regulation [89]. Mechanistically, circCTIC1 may bind to the NURF complex, allowing it to bind the c-Myc promoter and activate c-Myc transcription, leading to the self-renewal of TICs in colon cancer [89].

**Table 2:** Representative circRNAs and related signaling pathways in colon cancer

Circular RNA	Expression	Functions	Downstream targets	References
CircPPP1R12A	↑	Promotes tumorigenesis, Increases proliferation, migration, invasion	Hippo-YAP	[87]
CircPIP5K1A	↑	Increases viability, migration, invasion	miR-1273a, Zic-1, CDX-2, IRF-4	[88]
CircCTIC1	↑	Promotes tumorigenesis, self-renewal of stem cells	c-Myc	[89]
Circ_0001313	↑	Enhances radioresistance	miR-338-3p	[90]
CircRNA_104916	↓	Inhibits tumorigenesis, EMT, migration, invasion, induces apoptosis, controls cell cycle.	Apoptosis pathway, cyclin proteins	[91]
CircRNA-ACAP2	↑	Enhances proliferation, migration, invasion	hsa-miR-21-5p, Tiam1	[92]

The expression of circ_0001313, also known as circCCDC66, is increased in typical and radio-resistant colon cancer tissues. In both SW480 and SW620 cells, circ_0001313 expression is increased after irradiation treatment. Circ_0001313 knockdown enhances the radio-sensitivity of colon cancer cells and inhibits their viability and colony formation rate via regulation of miR-338-3p level [90]. CircRNA_104916 is downregulated in CRC tissues and is negatively associated with tumor size, T state and metastasis, and is correlated with a better survival in CRC patients. CircRNA_104916 upregulation stimulates apoptosis and cell cycle arrest at G2/M phase due to apoptosis pathway activation and increased cyclin proteins levels in LoVo and Caco-2 cells. Moreover, circRNA_104916 upregulation alleviates migratory and invasive ability of colon cancer cells via EMT suppression [91].

**Fig. 2:**
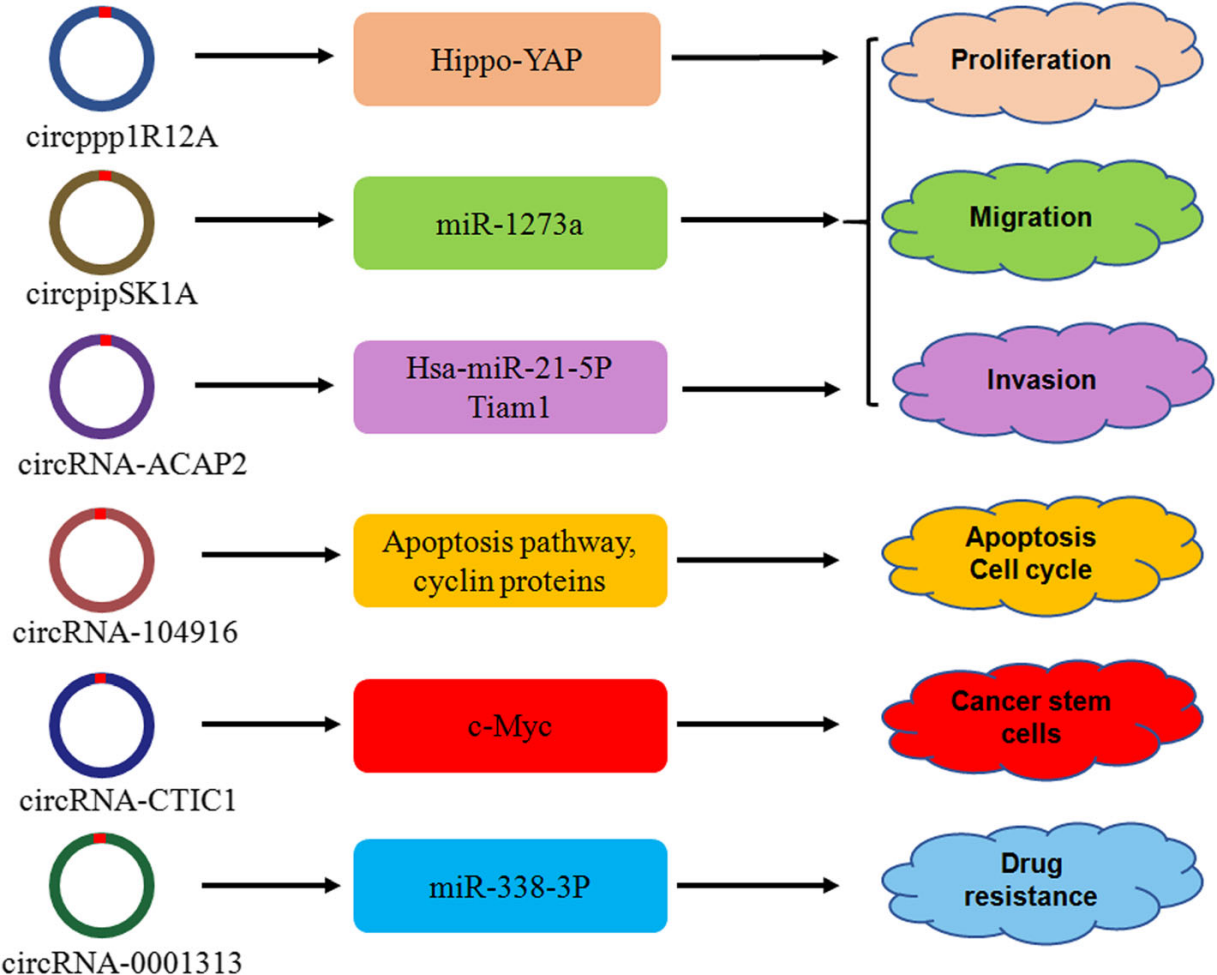
Multiple circular RNAs (circRNAs) are involved in colon oncogenesis and progression

The expression of circRNA-ACAP2 is higher in colon cancer tissues than in normal tissues, and its knockdown represses SW480 cell proliferation, migration and invasion via upregulation of miR-21-5p and further inhibition of Tiam1, a downstream target of miR-21-5p [92]. One group identified three circRNAs, including circ-CCDC66, circ-ABCC1 and circ-STIL, which are downregulated in the plasma of CRC patients. The level of circ-ABCC1 is associated with tumor growth and progression, and circCCDC66 and circ-ABCC1 levels are reduced in precursor lesions of CRC, indicating that these lncRNAs may be biomarkers for early stage CRC. The use of two traditional biomarkers of CRC, CEA and CA19–9, in combination with these three lncRNAs improves the diagnostic precision of CRC. Moreover, these three lncRNAs are useful for diagnosing CEA-negative and CA 19–9-negative CRC [93]. Thus, these circRNAs participate in colon tumorigenesis and progression (Fig. 2).

## Conclusions

In summary, lncRNAs and circRNAs have emerged as playing crucial roles in the initiation and progression of colon cancer. Targeting these noncoding RNAs could be helpful to obtain treatment benefit in colon cancer patients. Several compounds have been identified to regulate the expression of lncRNAs in human colon cancer [51, 94, 95]. For instance, ginsenoside Rg3 reduces the expression of lncRNA CCAT1 in colorectal cancer Caco-2 cells, leading to suppression of cell growth, migration and invasion [94]. Two anticancer agents, 3,3′-diindolylmethane and doxycycline, regulates several lncRNAs in HCT-116 and HT-29 colon cancer cells [51]. Different chemotherapeutic drugs, including 5-fluorouracil, oxaliplatin and irinotecan, elevated the expression of linc00973 in colon cancer HT-29 and HCT-116 cells [95]. In addition, identification of a stool lncRNAs panel might be useful for early detection of colorectal cancer [96].

However, several important questions must be addressed to obtain a full understanding the functions of lncRNAs in colon carcinogenesis. For instance, because lncRNAs can be multi-functional in regulating several cellular processes in various tissues, detailed molecular mechanisms of lncRNA functions needs to be appreciated on a case-by-case basis. Furthermore, it is necessary to validate whether the reliability and sensitivity of lncRNAs are enough for their clinical application as biomarkers. CEA and CA19–9 have been used in clinical colon cancer diagnosis, but whether lncRNAs and circRNAs have advantages compared these biomarkers need to be determined. We are optimistic that the use of new and robust sequencing technologies will elucidate roles of lncRNAs implicated in colon tumorigenesis may eventually accelerate the clinical application of lncRNAs for use in diagnosis, treatment, and prognosis evaluation.

## Data Availability

Not applicable.

## Abbreviations

AP-1: Activator protein 1
CA19–9: Carbohydrate antigen 19–9
CDX-2: Caudal type homeobox 2
CEA: Carcinoembryonic antigen
DHX36: DEAH box polypeptide 36
GSEC: G-quadruplex-forming sequence containing lncRNA
IRF-4: Interferon regulating factor 4
PRC2: Polycomb repressive complex 2
Tiam1: T lymphoma invasion and metastasis protein 1
TICs: Tumor-initiating cells
UHRF1: Ubiquitin-like plant homeodomain (PHD) and really interesting new gene (RING) Finger domain containing protein 1
UPAT: UHRF1 protein associated transcript
Zic-1: Zinc finger of the cerebellum 1
